# Polyneuritis Complicating Herpes Zoster

**Published:** 1964-07

**Authors:** E. S. Etuk, D. Bridges

**Affiliations:** University Department of Medicine, Bristol Royal Infirmary; University Department of Medicine, Bristol Royal Infirmary


### POLYNEURITIS COMPLICATING HERPES ZOSTER

BY

E. S. ETUK, M.B., CH.B. (BRISTOL), D.T.M.H. AND D. BRIDGES, M.B., CH.B. (BRISTOL)

*University Department of Medicine, Bristol Royal Infirmary*

Polyneuritis is a rare complication of herpes zoster. There are clinical details of only 13 cases in the literature. The following case is therefore presented and the aetiology of the neuropathy is discussed.

### CASE REPORT

A 47-year-old male bank clerk was admitted to the Bristol Royal Infirmary on 7th October 1963. On admission he gave a history of anorexia for 2 weeks and left sided upper abdominal pain for 1 week. Vesicles had been present over the painful area for 6 days. On the day before admission he awoke in the morning and found that he had lost the use of all his limbs. There were no bulbar symptoms.

In 1957 chronic lymphatic leukaemia had been diagnosed and since that time the patient had been regularly observed in the out-patient department. Treatment was initially with steroids because of haemolytic anaemia, and later in 1962 he had a course of chlorambucil. However, the only drug he had taken in the 7 months before the present admission was a maintenance dose of prednisone, 5 mgs b.d.

On examination, the classical eruption of herpes zoster was present over the left dermatome of T.10). In addition there were a few scattered vesicles over the trunk.

The mental state was normal, and the cranial nerves were intact. There was profound weakness of all limbs, maximal proximally. The only movements he was able to make were of the fingers. The limbs were hypotonic and tendon reflexes were absent; the plantar responses were unobtainable. Sensory loss was detected over a glove-and-stocking distribution. Marked hepato-splenomegaly was detected on abdominal palpation and there were also a few enlarged lymph nodes in the axillae.

*Investigations*

Haemoglobin: 12·9 g/100 ml.
W.B.C.: 11,400/cu. mm., 2,100 polymorphonuclear leucocytes.
C.S.F.: Fluid was turbid with a yellow tint. Pressure: 200 mms. C.S.F.; Cells: 50 round cells and 50 R.B.C.s/cu.mm.; Protein: 140 mgm/100 ml.; Glucose: 50 mgm/100 ml.; W.R. negative; Lange 5544433211.

A diagnosis of polyneuritis possibly due to herpes zoster complicating chronic lymphatic leukaemia was made. Treatment with prednisone was continued at 5 mgm b.d.

*Progress*

Recovery of the sensory defect began during the first few days after admission and was followed by improvement in power of the limbs. With graduated exercises improvement continued, so that 5 weeks after admission he was discharged home with full return of power in his arms and able to walk with the aid of a walking stick.

### DISCUSSION

Herpes zoster is a well recognized complication in patients with chronic lymphatic leukaemia. Acute polyneuritis, however, is a very rare development in patients with herpes zoster. Knox *et al.* (1961) reported 3 cases and reviewed details of 10 others in the literature. In these cases, the latent period between the eruption and the onset of the polyneuritis varied from a few days to several weeks. The suggestion was made that the length of the latent period was a good index of the prognosis, a short period (up to 2 weeks) being followed by a severe and fatal illness, while a long period was followed by a mild illness. This is not supported by the present case, since although the latent period was only 6 days and the patient was severely incapacitated, an excellent functional recovery has taken place.

The pattern of the neurological sequelae following herpes zoster varies from encephalomyelopathy to polyradiculoneuropathy with or without spinal cord involvement. The cause is uncertain; Knox *et al.* suggested that the widespread disorders came as a result of an "allergic" process such as that which complicates chicken pox and other exanthemata, and that the use of corticosteroids is therefore indicated. The other possibility is that the virus disseminates widely in the nervous system in some cases. The role that corticosteroids may have played in the dissemination of the lesions in our case is uncertain but it is possible that the spread was facilitated by the prednisone. Some evidence for such a spread of the virus, rather than an allergic response, is afforded by the scattered vesicles which occurred over the trunk. However, in another respect the corticosteroids may have been helpful since of the thirteen previously reported patients 7 died; only 3 were given corticosteroids and 2 of these recovered. It is tempting to suggest that the "allergic" response was modified in our patient, since at the time of infection he was already taking corticosteroids which were continued throughout the illness.

### SUMMARY

A case of herpes zoster complicated by "polyneuritis" is described. The patient was known to have chronic lymphatic leukaemia. Profound limb muscle weakness and sensory loss was present but good recovery ensued. The role of corticosteroids in this case is discussed.

*Acknowledgements*

We are grateful to Professor C. Bruce Perry for permission to publish this case which was under his care, and to Dr. K. R. Gough for his encouragement and advice.

### REFERENCE

Knox, J. D. E., Levy, R. and Simpson, J. A. (1961). *J. Neurol. Neurosurg. Psychiat.* **24**, 167.

